# Replacement Effect of Fish Meal by Plant Protein Sources in Olive Flounder (*Paralichthys olivaceus*) Feeds with an Addition of Jack Mackerel Meal on Growth, Feed Availability, and Biochemical Composition

**DOI:** 10.1155/2023/7965258

**Published:** 2023-07-15

**Authors:** Seong Il Baek, Hae Seung Jeong, Sung Hwoan Cho

**Affiliations:** ^1^Department of Convergence Study on the Ocean Science and Technology, Korea Maritime and Ocean University, Busan 49112, Republic of Korea; ^2^Advanced Aquaculture Research Center, National Institute of Fisheries Science, Changwon-si 51688, Gyeonsangnam-do, Republic of Korea; ^3^Division of Marine Bioscience, Korea Maritime and Ocean University, Busan 49112, Republic of Korea

## Abstract

Application of feed stimulants is very helpful to increase the feed intake of fish, especially in the development of low fish meal (FM) diets. FM replacement effect by various plant protein sources (corn gluten meal (CGM), soy protein concentrate (SPC), and corn protein concentrate (CPC)) in diets with an addition of jack mackerel meal (JMM) as feed stimulants on growth, feed availability, and biochemical composition of olive flounder was elucidated. An experimental design of two-way (two replacement levels (25% and 50%) × 3 replacement sources (CGM, SPC, and CPC)) analysis of variance was adopted. Seven diets were formulated. Amount of 60% FM was contained in the control (Con) diet. In the Con diet, 25% and 50% FM were replaced by CGM, SPC, and CPC with an addition of 12% JMM as feed stimulants, referred to as the CGM25, CGM50, SPC25, SPC50, CPC25, and CPC50 diets, respectively. Four hundred and twenty juvenile fish were distributed into 21 flow-through tanks. All diets were assigned to triplicate groups of fish. Fish were hand-fed to satiation twice a day for 56 days. Both dietary replacement levels and sources had statistical effect on weight gain (*P* < 0.0001 and *P* < 0.045, respectively), specific growth rate (SGR) (*P* < 0.0001 and *P* < 0.033), and feed consumption (*P* < 0.0001 and *P* < 0.03) of fish. Dietary increased FM replacement levels lowered weight gain, SGR, and feed consumption of fish. Weight gain, SGR, and feed consumption of fish fed the Con and CGM25 diets were statistically (*P* < 0.05) greater than those of fish fed the CGM50, SPC50, and CPC50 diets. Both replacement level and source had no statistical effect on feed utilization, biochemical composition except for statistical effect of replacement source on glycine content of fish, and lysozyme and superoxide dismutase (SOD) activities of fish. FM up to 25% could be substituted with CGM, SPC, and CPC in the olive flounder feeds supplemented with 12% JMM as feed stimulants without compromising growth, feed utilization, and lysozyme and SOD activities.

## 1. Introduction

Olive flounder (*Paralichthys olivaceus*) is one of the most commercially valuable marine flatfish in the Eastern Asia countries, such as the Republic of Korea (*hereafter*, Korea), China, and Japan due to its good taste, rapid growth, high market value, and eurythermal endurance [1, 2]. Annual aquaculture production of olive founder in Korea dramatically increased from 14,127 metric tons in 2,000–41,791 metric tons in 2021, ranked the highest in aquaculture production and economic value (USD 465 million) among culturable marine fish species (89,436 metric tons and USD 847 million, respectively) in the same year [3]. Increased aquaculture production of olive flounder needs high amount of feed, and in Korea, there is still higher preference for the moist pellet (MP) by farmers rather than the formulated feed (FF) [4]. Higher amount (243,053 metric tons) of the MP than the FF (19,293 metric tons) was used for olive flounder culture in Korea in 2021 [3]. Application of the MP in fish culture increased discharged wastes, and storage and fish production costs as well as lowered fish growth over the EP [5–7]. Thus, adoption of the EP is highly recommended for sustainably intensive fish culture globally to resolve those challenges.

The commercial feeds for olive flounder culture commonly include approximately 60% of fish meal (FM) [8], which is not sustainable and inexpensive because of the dwindled or stagnant supply of FM. There is, thus, a high request for scientists to look for a replacer for FM in fish feeds, and plant-derived protein source is one of the replacers for FM in fish feeds. Several studies have proved plant protein sources, such as corn gluten meal (CGM), defatted soybean meal, and soy protein concentrate (SPC), as a single suitable alternative to FM in olive flounder feeds [9–11]. Since those plant protein sources are produced in higher quantities and often less expensive than FM, their expanded use in fish feeds does not threaten the overexploitation of limited resources [12]. Nevertheless, the substitutability of alternative plant protein sources for FM is frequently restricted by several factors, such as deficiency or imbalance of essential amino acids (EAA) and/or essential fatty acids, presence of antinutritional factors (ANF) or toxins, and deteriorated palatability by fish [13]. Some of these limitations can be resolved by application of optimal combinations of different plant protein sources to balance essential nutrient profiles, and lower ANF (for instance, heat treatment to inactivate heat-labile components) or limit their optimum inclusion level in diet that does not lead to deteriorated growth of fish [14].

Several processing technologies have been developed to minimize ANF and carbohydrates but increase the protein content of plant products. Protein concentrates have been prepared from a variety of plant-source meals, including soybean, canola, corn, wheat, and pea. CGM is the residue from corn after removing all starch and germ and after the separation of the bran. It is regarded as a good source of methionine but a relatively poor source of lysine [12]. Numerous studies have suggested CGM as a suitable replacer to FM in several fish feeds [11, 15–19]. Among plant protein sources, soybean products have received attention because of their low cost and consistent supply and quality. SPC is a product through aqueous ethanol or methanol extracting of solvent-extracted soybean meal, and some ANF were inactivated through extraction process [17]. Lim and Akiyama [21] highlighted that from a nutritional, economic, and market availability standpoints, soybean products, including full-fat soybean meal, soybean meal, and SPC, have the potential to be the key ingredient in the future aquafeed industry. Partial substitution of FM by SPC in some fish diets were made without compromising growth performance [17, 22–24]. Corn protein concentrate (CPC) is a manufactured corn protein prepared by enzymatic removal of non-protein components of corn [25]. Shekarabi et al. [26] proved that dietary replacement of FM by CPC at 9% produced comparable growth to rainbow trout (*Oncorhynchus mykiss*) fed a 45% FM-basal diet. Replacement of FM up to 50% and 53.4% with CPC could be made in red hybrid tilapia (*Orechromis* sp.) and tilapia (*Oreochromis niloticus*) feeds, respectively without any undesirable effect on growth [25, 27]. In addition, Ng et al. [27] emphasized that inclusion of palatability enhancer (feed attractant) elevated growth of tilapia fed a plant protein concentrate-basal diet. Thus, substitutability of plant protein source for FM in fish diets could be improved by inclusion of feed stimulants (enhancer).

Feed attractants and/or stimulants are the critical component of food for several fish species in wild, and they contain free AA, betaine, nucleosides, nucleotides, organic acids, and quaternary ammonium compounds [28, 29]. Nevertheless, the effectiveness of incorporated synthetic chemicals of feed attractants and/or stimulants in diets on the growth of fish in practical feeding is still controversial [29, 30]. Inclusion of crude jack mackerel meal (JMM) exhibiting the highest attractiveness to olive flounder among 15 crude feed ingredients at 5% in EP achieved dramatic improvement in growth directly responded from improved feed consumption [31]. Later, the dietary optimum inclusion level of JMM was reported to be at 12% for the best growth and highest feed consumption of olive flounder [32]. In the study of Jeong and Cho [33], FM up to 25, and 50% could be substituted by meat meal and chicken and tuna byproduct meals, respectively in olive flounder diets supplemented with 12% JMM as feed stimulants without compromising growth.

Manipulation of feed ingredients to incite strong attractiveness to target fish in low FM feed can be the very sustainable fish culture technique to improve the growth of fish responded from increased feed consumption. Therefore, we aim to determine the replacement effect of FM by alternative plant protein sources (CGM, SPC, and CPC) in diets supplemented with JMM as feed stimulants on growth, feed utilization, biochemical composition, hematological parameters, and innate immune response of juvenile olive flounder.

## 2. Materials and Methods

### 2.1. Rearing Conditions of the Feeding Experiment

Juvenile fish were bought from Hyun fish hatchery (Chungcheongnam-do, Korea) for the feeding trial. Prior to the start of the feeding experiment, fish were acclimated to rearing conditions by feeding with EP (55% crude protein and 8% crude lipid) (Suhyup Feed, Gyeongsangnam-do, Korea) for 14 days. A total of 420 juvenile fish averaging 18 g were equally distributed into 21, 50 L flow-through tanks (20 fish per tank). Sand-filtered seawater and proper aeration were supplied to each tank. The flow rate of seawater was 3.5 L/tank/min. Water conditions of the experimental tanks were monitored by using a digital multimeter (AZ-8603, AZ Instrument, Taiwan) as the followings: temperature range of 17.5–24.0°C (21.2 ± 1.95°C; mean ± SD), dissolved oxygen range of 7.0–7.9 mg/L (7.2 ± 0.12 mg/L), pH range of 7.1–7.5 (7.3 ± 0.10), and salinity range of 31.6–33.6 g/L (32.6 ± 0.47 g/L). Fish were carefully hand-fed to apparent satiation twice (08:30 and 16:30) daily for 56 days. Uneaten feeds were not collected.

### 2.2. Design of the Feeding Trial

An experimental design of two-way (two replacement levels (25% and 50%) × 3 replacement sources (CGM, SPC, and CPC)) analysis of variance (ANOVA) was adopted. In total, seven experimental diets were prepared (Table 1). The amount of 60% FM (the blend of sardine and anchovy meal = 1 : 1, as is) and 15% fermented soybean meal were contained as the protein sources in the control (Con) diet. Wheat flour at 18.3% and fish and soybean oils at 4.2% were included as the carbohydrate and lipid sources, respectively, in the Con diet. In the Con diet, 25% and 50% FM were replaced by CGM, SPC, and CPC, with an addition of 12% JMM producing the best weight gain of olive flounder [34] at the expense of FM, referred to as the CGM25, CGM50, SPC25, SPC50, CPC25, and CPC50 diets, respectively. All diets were formulated to be isonitrogenous at 56.0% and isolipidic at 10.5%. Dietary protein and lipid content were all met for the requirements of juvenile olive flounder [34, 35].

The ingredients of the experimental diets were blended well, and water was added to the mixture at a ratio of 3 : 1 to obtain the preferred consistency. The blend was pelletized by using a laboratory pellet extruder (Dongsung Mechanics, Busan Metropolitan City, Korea). Finally, the pelletized diets were then air-dried at 40°C in an electronic dry machine (UDS-4522F, Kyung Dong Navien, Pyeongtaek-city, Gyeonggi-do, Korea) for a day and kept at –20°C until use.

### 2.3. Evaluation of the Biological Indices of Olive Flounder

After the 56-day feeding experiment, live fish were starved for a day and then anesthetized by tricaine methanesulfonate (MS-222) at 100 ppm. All surviving fish from each tank were counted and weighed collectively. Randomly chosen 10 fish from each tank were used to calculate the biological indices. Growth measurements and biological indices of olive flounder were evaluated as the followings; specific growth rate (SGR, %/day) = ((Ln final weight of fish – Ln initial weight of fish) × 100)/days of feeding trial, feed efficiency ratio (FER) = weight gain of fish/feed consumption, protein efficiency ratio (PER) = weight gain of fish/protein consumption, protein retention (PR, %) = protein gain of fish × 100/protein consumption, condition factor (CF, g/cm^3^) = total weight of fish (g) × 100/total length of fish (cm)^3^, viscerosomatic index (VSI, %) = viscera weight × 100/total weight of fish, and hepatosomatic index (HSI, %) = liver weight × 100/total weight of fish.

### 2.4. Hematological Parameters of Olive Flounder

The blood samples were taken with the heparinized syringes from the caudal veins of three anesthetized fish from each tank. Then the plasma was separated by centrifugation at 2,716 × *g* at 4°C for 10 min and kept at –70°C for analysis of aspartate aminotransferase (AST), alanine aminotransferase (ALT), alkaline phosphatase (ALP), total bilirubin (TBL), total cholesterol (TCL), triglyceride (TGL), total protein (TPT), and albumin (ABM) by using an automatic chemistry system (Fuji Dri-Chem NX500i, Fujifilm, Tokyo, Japan).

### 2.5. Analysis of Innate Immune Response of Olive Flounder

Blood of three fish from each tank was extracted from the caudal fin after the feeding trial. Serum from the blood samples was separated by centrifugation at 2,716 × *g* at 4°C for 10 min and kept at –70°C. The turbidimetric assay for lysozyme activity was performed based on Lange et al.'s [36] study. Superoxide dismutase (SOD) activity was measured using a SOD ELISA kit (MyBioSource, mbs705758) to evaluate the ability of the test solution to inhibit the reaction of superoxide. The same methods and procedures for measuring lysozyme and SOD activities of the fish were used in Jeong et al.'s [32] study.

### 2.6. Analysis of Biochemical Composition of the Samples

Fifteen fish at the start of the feeding experiment and all remaining four fish from each tank at the end of the 56-day feeding experiment were sampled for the biochemical composition analysis. The moisture, crude protein, crude lipid, and ash content were carried out based on the standard AOAC procedures [37]. All AA in the experimental diets and the whole body of olive flounder were analyzed by using an AA analyzer (L-8800 Auto-analyzer: Hitachi, Tokyo, Japan), followed by an ion-exchange chromatography after hydrolyzing with 6N HCl at 110°C for 24 hr. FA were identified by comparing the experimental diets and whole-body fish with that of known standards (37 component FAME mix; Supelco™, St. Louis, MO, USA). Lipids for FA analyses in the experimental diets and whole-body fish were extracted by a mixture of chloroform and methanol (2 : 1 v/v), according to Folch et al. [38]. The same methods and procedures for the biochemical composition of the experimental diets and fish were used in Jeong et al.'s [32] study.

### 2.7. Statistical Analysis

Significant differences in dietary treatment means were analyzed at *P* = 0.05 by two-way ANOVA and Tukey's honestly significant difference test by using SPSS version 24.0 (SPSS Inc., Chicago, IL, USA). Percentage data were arcsine-transformed for statistical analysis.

## 3. Results

### 3.1. AA and FA Profiles of the Experimental Diets

CGM and SPC included lower EAA content, except for leucine, methionine, and phenylalanine, and arginine and phenylalanine, compared to FM, respectively (Table 2). CPC also included lower EAA content, except for isoleucine, leucine, methionine, and phenylalanine, compared to FM. Arginine (3.21%–4.34% of diet) and lysine (3.43%–5.56% of diet) content in all diets satisfied their dietary requirements for olive flounder, but not methionine requirement in the Con, SPC25, and SPC50 diets.

Eicosapentaenoic acid (EPA) and docosahexaenoic acid (DHA) were not detected in all plant replacement sources (CGM, SPC, and CPC) for FM (Table 3). The sum of *n*−3 highly unsaturated FA (∑*n*−3 HUFA) content in FM was higher than that in CGM, SPC, and CPC. Elevated levels of FM replacement by CGM, SPC, and CPC resulted to decreased sum of saturated FA (∑SFA) and ∑*n*−3 HUFA content in all CGM, SPC, and CPC substituted diets. Elevated FM replacement levels by CGM and SPC in the diets led to an increased sum of monounsaturated FA (∑MUFA). The content of ∑*n*−3 HUFA in the CGM25, CGM50, SPC50, CPC25, and CPC50 diets seemed to be lower than dietary *n*−3 HUFA requirement for olive flounder.

### 3.2. Performance of Olive Flounder in the 56-Day Feeding Experiment

All fish survived at the end of the 56-day feeding experiment (Table 4). Weight gain (*P* < 0.0001 and *P* < 0.045) and SGR (*P* < 0.0001 and *P* < 0.033) of fish were statistically altered by both dietary replacement level and source, respectively. Dietary increased FM replacement levels lowered weight gain and SGR of fish. Weight gain and SGR of fish fed the Con and CGM25 diets were statistically (*P* < 0.05) greater than those of fish fed CGM50, SPC50, and CPC50 diets but not statistically different from those of fish fed the SPC25 and CPC25 diets.

Feed consumption of fish was statistically altered by both replacement levels (*P* < 0.0001) and sources (*P* < 0.03) as well as their interaction (*P* < 0.012) (Table 5). Dietary increased FM replacement levels lowered the feed consumption of fish. Feed consumption of fish fed the Con and CGM25 diets were statistically (*P* < 0.05) higher than that of fish fed the CGM50, SPC50, and CPC50 diets but not statistically (*P* > 0.05) different from that of fish fed the SPC25 and CPC25 diets. Neither dietary replacement level nor replacement source, except for statistical (*P* < 0.037) effect of dietary replacement level on VSI statistically (*P* > 0.05) alter FER, PER, PR, CF, VSI, and HSI of fish. Nevertheless, no statistical difference in VSI of fish was found among dietary treatments.

### 3.3. Biochemical Composition of the Whole-Body Olive Flounder

Moisture content of the whole-body fish varied from 70.9% to 71.7%, crude protein varied from 18.0% to 18.4%, crude lipid varied from 3.2% to 3.7%, and ash content varied from 3.7 to 4.4 were not statistically (*P* > 0.05) altered by either replacement level or replacement source, except for statistical (*P* < 0.035) effect of the interaction of replacement level and source on crude protein content (Table 6). Nevertheless, no statistical difference in crude protein content of the whole-body fish was found among dietary treatments.

AA (Table 7) and FA (Table 8) profiles of the whole-body olive flounder were not statistically (*P* > 0.05) influenced by either replacement level or replacement source, except for the statistical (*P* < 0.024) effect of replacement source on glycine content.

### 3.4. Hematological Parameters of Olive Flounder

Hematological measurements of fish were not statistically (*P* > 0.05) influenced by either replacement level or replacement source, except for statistical (*P* < 0.008) effect of replacement source on TCL (Table 9).

### 3.5. Innate Immune Response of Olive Flounder

Both dietary replacement level and source had no statistical (*P* > 0.05) effect on lysozyme activity varied from 381.6 to 450.4 U/mL, and SOD activity of fish varied from 71.5% to 73.6% (Table 10).

## 4. Discussion

No distinctive differences in weight gain and SGR of olive flounder fed the Con, CGM25, SPC25, and CPC25 diets in the current study indicated that 25% FM could be substituted by CGM, SPC, and CPC in olive flounder diets with an addition of 12% JMM as feed stimulants without deteriorating growth performance. Kikuchi [11] revealed that weight gain of olive flounder fed diets substituting FM up to 40% by CGM with AA supplementation was comparable to fish fed a 75% FM-basal diet when juvenile fish were fed with a 75% FM-basal diet or diets substituting 20%, 40%, and 60% FM by CGM with AA (arginine, lysine and tryptophan) supplementation or 40% FM by CGM without AA supplementation. However, in this study, weight gain and SGR of olive flounder fed a diet replacing 40% FM by CGM without AA supplementation were poorer than those of fish fed a 75% FM diet or diet replacing 40% FM by CGM with AA supplementation. Replacement of FM up to 60% by CGM in the spotted rose snapper (*Lutjanus guttatus*) diets supplemented with lysine, arginine, and CaHPO_4_ led to comparable growth to fish fed a 55% FM-basal diet [15]. Chen et al. [22] suggested that limited FM replacement (less than 30%) should be made in diets when juvenile pearl gentian grouper (*Epinephelus lanceolatus* × *E. fuscoguttatus*) were fed with a 65% FM-basal diet or diets substituting 15%, 30%, 45%, 60%, and 75% FM by CGM. Nandakumar et al. [17] proved that dietary FM substitution up to 29% by CGM could be made without compromising the growth of fish and digestibility when juvenile Asian seabass (*Lates calcarifer*) were fed with a 35% FM-basal diet or diets substituting 15%, 29%, 44%, and 59% FM by CGM for 45 days.

Weight gain of fish fed any SPC diet replaced for FM was inferior to olive flounder fed a 74% FM-basal diet when juvenile (initial weight of 2.45 g) fish were fed with a 74% FM basal diet or diets substituting 25%, 50%, 75%, and 100% FM by SPC or 75% FM by SPC with AA supplementation for 9 weeks [9], but they also suggested that AA supplementation in low FM diet was able to improve growth and feed intake. In considering comparable growth performance of olive flounder fed the SPC25 diet to fish fed the Con diet in the current study versus poorer weight gain of the same fish species fed any SPC diet replaced for FM than that of fish fed a 74% FM-basal diet in Deng et al.'s [9] study, substitutability of SPC for FM in the olive flounder diet with inclusion of 12% JMM as feed stimulants could be improved from 0% to 25% without AA supplementation. Weight gain of juvenile rice field eel (*Monopterus albus*) fed a diet replacing 60% FM by SPC was similar to that of fish fed a 55% FM-basal diet [17]. However, FM replacement up to 82.5% by SPC in diets supplemented with lysine and methionine could be successfully made without any detrimental the effect on growth of black seabream (*Acanthopagrus schlegelii*) [24]. They also emphasized that SPC based diet supplemented with phytase could lower phosphorous load in aquatic surroundings. Day and GonzÁlez [23] also revealed that 25% FM substitution by SPC in diet supplemented with methionine produced similar growth to turbot fed a 70.5% FM-basal diet.

Ng et al. [27] unveiled that 50% FM replacement by CPC could be made in a 48% FM-basal diets of red hybrid tilapia without causing any detrimental effect on growth, FER, and PER. Later, Ng et al. [39] highlighted that the inclusion of feed attractants (betaine-HCl) at 5% in low FM diet replacing 75% FM with the combined 50% CPC and 25% SPC produced comparable growth, and SGR to red hybrid tilapia fed a 47% FM-basal diet. Likewise, Khosravi et al. [40] explained that incorporated various hydrolysates to low FM diets substituting 50% FM by the combined SPC and CGM produced comparable growth to olive flounder fed a 55% FM-basal diet, but higher compared to fish fed a low FM diet without any hydrolysate inclusion when juvenile fish were fed with a 55% FM-basal diet or diets substituting 50% FM with the combined SPC and CGM supplemented with lysine, methionine, and taurine without any hydrolysate or with the inclusion of various (shrimp, tilapia, and krill) hydrolysates for 11 weeks.

Thirty percent FM substitution with fermented plant-based protein sources (soybean meal : CGM = 1 : 1) in diets supplemented with lysine and methionine produced comparable growth to olive flounder fed a 70% FM-basal diet, but further increased FM replacement (40%) led to poorer growth [41]. Dietary FM replacement up to 40% by the combined plant protein sources produced comparable growth to growth-out olive flounder fed a 65 FM-basal diet when fish were fed with a 65% FM-basal diet or diets substituting 25%, 30%, 35%, and 40% FM by the plant protein sources (soybean meal, SPC, and wheat gluten) with AA (lysine, threonine, methionine, and taurine) supplementation for 15 weeks [42]. Therefore, the substitutability of plant protein source for FM in fish diets seems to vary profoundly relying on the type and kind of an alternative plant protein source, FM replacement level, with or without supplementation of AA that are likely to be lack or deficiency in a selected plant source, and with or without the inclusion of feed stimulants (enhancer).

Looking for an appropriate substitute for FM is crucial for reducing sustainability issues related to FM usage in fish feeds [43]. All experimental diets satisfied arginine (2.04%–2.10% of diet) and lysine (1.55%–1.97% of diet) requirements for juvenile olive flounder [44, 45] in the current study, while methionine content of the CGM25, CGM50, CPC25, and CPC50 diets satisfied dietary requirement (1.44%–1.49% in the presence of 0.06% cysteine) of olive flounder [46]. Methionine requirement in diets might be spared by cysteine to some extent (40%–50%) in red drum (*Sciaenops ocellatus*) and yellow perch (*Perca flavescens*) [47, 48]. Since the sum of methionine and cysteine content in the Con, SPC25, and SPC50 diets were 2.37%, 2.07%, and 1.66% of the diet, respectively, which were higher than their sum (1.50%–1.55% in diet) suggested by Alam et al. [46], their methionine content did not seem to deteriorate growth of olive flounder. Nevertheless, the SPC50 diet containing the lowest methionine content led to the poorest growth of fish.

In many marine fish diets, ∑*n*−3 HUFA, such as DHA and EPA, is a critical factor for the survival and normal growth of fish [49–53]. The Con and SPC25 diets fulfilled dietary ∑*n*−3 HUFA requirement (7.62%–9.52% of total FA) for olive flounder [53] in the current study. High FM replacement levels from 25% to 50% by alternative plant sources (CGM, SPC, and CPC) led to reduced weight gain of fish in the current study, probably resulted from decreased ∑*n*−3 HUFA content in high FM-replaced diets.

Fish-fed diets substituting FM to higher than proper limit with alternative protein sources commonly led to poorer growth due to reduced feed consumption, resulted from deteriorated palatability [54, 55]. No remarkable difference in feed intake of olive flounder fed the Con and diets replacing 25% FM by plant protein source (CGM25, SPC25, and CPC25) diets, but higher than that of fish fed diets replacing 50% FM by plant protein sources in the current study might indicate that 12% JMM inclusion in diets replacing 25% FM with all plant protein sources enhanced feed consumption, and eventually led to comparable growth to fish fed the Con diet. Reduction in feed consumption of olive flounder-fed diets substituting 50% FM by all plant protein sources could be partially demonstrated by lower histidine content in the 50% FM replaced diets compared to that in the 25% FM replaced diets based on Ikeda et al.'s [28] study emphasizing that histidine exhibited the strongest feeding activity for olive flounder among the synthetic AA of the extracts of jack mackerel. Likewise, elevated growth of olive flounder-fed diets replacing 50% FM by the combined SPC and CGM with various hydrolysates was closely associated with elevated feed consumption [40]. Dietary 40% FM substitution by the blend of three plant protein sources with AA supplementation did not deteriorate the feed intake of olive flounder and eventually led to comparable growth to fish fed a 65% FM-basal diet [42]. Kim et al. [56] also highlighted that the blend of plant protein sources can substitute FM up to 30% in the diets of olive flounder without causing any detrimental effect on growth and feed intake when juvenile fish were fed with a 65% FM-basal diet or diets substituting 10%, 15%, 20%, 25%, and 30% FM by the plant protein sources (soybean meal, wheat gluten, and SPC) with AA (lysine, methionine, and threonine) supplementation.

Both dietary replacement level and source had no remarkable effect on feed utilization or biological indices of olive flounder, except for the significant effect of dietary replacement level on VSI in the current study. Growth performance of olive flounder was directly responded from feed consumption, and it resulted in no difference in feed utilization. Likewise, dietary FM substitution with various protein sources did not alter feed utilization [33, 57–59] or biological indices of fish [33, 57]. Ng et al. [27] also proved that 50% FM substitution by CPC in diet did not influence feed utilization (FER and PER), biological indices, intraperitoneal fat, or gonadosomatic index of red hybrid tilapia. No difference in FER and PER was also found among diets when olive flounder were fed with a 65% FM-basal diet or diets substituting 25%, 30%, 35%, and 40% FM by the combined plant protein sources with AA supplementation [42]. Dietary substitution of FM by CGM altered feed utilization of spotted rose snapper and Asian seabass but did not have biological indices [15, 17].

The biochemical composition (proximate composition, and AA and FA profiles) of the whole-body fish, except for the significant effect of replacement source on glycine, was not altered by dietary treatments in the current study. Likewise, the biochemical composition of the whole body of olive flounder was not influenced by either dietary replacement level or dietary replacement source [33]. The proximate composition of the whole body of fish was not influenced by dietary FM substitution by various plant protein sources [17, 40–42]. Unlike these studies, the proximate composition of the whole-body fish was influenced by dietary FM replacement by various animal or plant protein sources [9, 11, 15, 17, 22, 24, 27, 39, 55, 60–62]. Dietary FM substitution with plant or animal protein sources influenced the AA profiles of the whole-body fish [9, 22, 60].

Hematological measurements of fish have been regarded as the critical evidences to evaluate physiological, health and nutritional status, and welfare of fish [63, 64]. In this study, no remarkable difference in the hematological measurements of fish was found among dietary treatments. Dietary FM replacement by various alternative sources did not influence serum chemistry [42, 62] or hematological parameters of olive flounder except for ALT [40]. Some contracting studies demonstrating that dietary FM replacement by various protein sources altered the hematological parameters of olive flounder [11, 65, 66], red sea bream (*Pagrus major*) [67], rice field eel [20], and spotted rose snapper [15] were also reported.

Fish use their innate immune system to protect themselves from infection [68–70]. Lysozyme and SOD activities are some of these innate immune responses of fish. The mucolytic enzyme (lysozyme) helps to fight off microbes in fish body [69, 71], and SOD is an antioxidant enzyme, which defends against other oxidizing activities that may affect fish [72]. No distinctive differences in lysozyme and SOD activities of fish were found among dietary treatments in the current study, probably demonstrating that dietary 25% and 50% FM replacements by CGM, SPC, and CPC did not deteriorate innate immunity of olive flounder. Likewise, dietary FM replacement by plant protein (soybean meal and CGM) or animal protein sources did not influence the innate immune response of olive flounder [33, 41]. Unlike these studies, however, dietary FM replacement by fermented soybean meal elevated SOD and glutathione peroxidase activities of rockfish [73]. Khosravi et al. [40] explained that 50% FM substitution by the combined SPC and CGM in diet supplemented with various hydrolysates affected innate immune responses of olive flounder, including nitroblue tetrazolium activity, total immunoglobulin, lysozyme activity, and SOD activity, and survival of fish infected with *Edwardsiella tarda*. Feasibility of FM substitution effect with CGM, SPC, and CPC in the diets of olive flounder with an addition of JMM as feed stimulants in practical feeding trial is needed.

## 5. Conclusion

Various plan protein sources (CGM, SPC, and CPC) could substitute FM up to 25% in the olive flounder feeds supplemented with 12% JMM as feed stimulants without compromising growth, feed intake, feed utilization, biochemical composition, and lysozyme and SOD activities. However, further increased FM substitution (50%) in diet deteriorated the growth and feed consumption of fish.

## Figures and Tables

**Table 1:** Ingredient and chemical composition of the experimental diets (%, DM basis).

	Experimental diets						
	Con	CGM25	CGM50	SPC25	SPC50	CPC25	CPC50
*Ingredient* (*%*)							
Fish meal (FM)^a^	60.0	33.0	18.0	33.0	18.0	33.0	18.0
Corn gluten meal (CGM)^b^		16.6	33.2				
Soy protein concentrate (SPC)^b^				18.2	36.4		
Corn protein concentrate (CPC)^b^						13.8	27.6
Jack mackerel meal (JMM)^c^		12.0	12.0	12.0	12.0	12.0	12.0
Fermented soybean meal	15.0	15.0	15.0	15.0	15.0	15.0	15.0
Wheat flour	18.3	15.4	12.5	13.8	9.3	18.7	19.1
Fish oil	2.1	3.4	4.7	3.4	4.7	2.9	3.7
Soybean oil	2.1	2.1	2.1	2.1	2.1	2.1	2.1
Vitamin premix^d^	1.0	1.0	1.0	1.0	1.0	1.0	1.0
Mineral premix^e^	1.0	1.0	1.0	1.0	1.0	1.0	1.0
Choline	0.5	0.5	0.5	0.5	0.5	0.5	0.5
*Nutrients* (*%*, *DM*)							
Dry matter	97.2	96.6	96.6	97.4	97.2	96.7	96.9
Crude protein	56.1	56.2	55.9	56.0	56.2	55.9	56.0
Crude lipid	10.3	10.3	10.9	10.2	10.5	10.3	10.2
Ash	11.4	11.4	11.4	11.4	11.3	11.6	11.6

^a^Fish meal (FM) (crude protein: 73.3%, crude lipid: 8.6%, ash: 15.0%) was the blend of sardine and anchovy meal at the ratio of 1 : 1 (as is). ^b^Corn gluten meal (CGM) (crude protein: 69.4%, crude lipid: 1.0%, ash: 2.5%), soy protein concentrate (SPC) (crude protein: 64.5%, crude lipid: 0.4%, ash: 6.1%) and corn protein concentrate (CPC) (crude protein: 79.3%, crude lipid: 2.8%, ash: 1.0%) were purchased from Thefeed Co. Ltd. (Busan Metropolitan City, Korea). ^c^Jack mackerel meal (JMM) (crude protein: 73.8%, crude lipid: 9.1%, ash: 13.3%) was purchased from Daekyung Oil & Transportation Co. Ltd. (Busan Metropolitan City, Korea). ^d^Vitamin premix contained the following amount, which was diluted in cellulose (g/kg mix): L-ascorbic acid, 200; *α*-tocopheryl acetate, 20; thiamin hydrochloride, 5; riboflavin, 8; pyridoxine, 2; niacin, 40; Ca-D-pantothenate, 12; myo-inositol, 200; D-biotin, 0.4; folic acid, 1.5; p-amino benzoic acid, 20; K_3_, 4; A, 1.5; D_3_, 0.003; cyanocobalamin, 0.003. ^e^Mineral premix contained the following ingredients (g/kg mix): NaCl, 7, MgSO_4_·7H_2_O, 105; NaH_2_PO_4_·2H_2_O, 175; KH_2_PO_4_, 224; CaH_4_(PO_4_)_2_·H_2_O, 140; ferric citrate, 17.5; ZnSO_4_·7H_2_O, 2.8; Ca-lactate, 21.8; CuCl, 0.2; AlCl_3_·6H_2_O, 0.11; KIO_3_, 0.05; Na_2_Se_2_O_3_, 0.007; MnSO_4_·H_2_O, 1.4; CoCl_2_·6H_2_O, 0.07.

**Table 2:** Amino acids profiles (% of the diet) of the experimental diets.

	FM	JMM	CGM	SPC	CPC	Requirement	Experimental diets						
							Con	CGM25	CGM50	SPC25	SPC50	CPC25	CPC50
*Essential amino acid* (*EAA*, *%*)													
Arginine	4.08	4.19	2.15	4.55	1.99	2.04–2.10^a^	3.37	3.18	3.09	3.43	3.56	3.14	3.05
Histidine	1.69	3.03	1.32	1.61	1.35		1.24	1.37	1.31	1.43	1.40	1.41	1.36
Isoleucine	2.69	2.90	2.44	2.63	2.07		2.07	2.06	2.02	2.13	2.10	2.15	2.16
Leucine	5.01	5.29	10.95	4.86	12.7		4.97	5.59	5.82	5.03	4.90	5.74	6.00
Lysine	5.56	5.96	1.22	4.14	0.93	1.55–1.97^b^	3.86	3.44	3.19	3.78	3.59	3.37	3.05
Methionine	1.13	2.10	1.75	0.29	1.80	1.44–1.49^c^	1.25	1.29	1.35	1.20	1.16	1.32	1.37
Phenylalanine	2.78	2.86	4.02	3.11	4.48		2.35	2.47	2.59	2.39	2.43	2.52	2.64
Threonine	3.10	3.28	2.28	2.54	2.44		2.30	2.20	2.10	2.22	2.13	2.24	2.16
Tryptophan	1.14	1.22	0.28	0.64	0.29		0.49	0.40	0.34	0.45	0.37	0.42	0.35
Valine	3.37	3.54	2.92	2.85	2.59		2.60	2.55	2.49	2.50	2.43	2.63	2.58
∑EAA	3055	34.37	29.33	27.22	30.64		24.50	24.55	24.30	24.56	24.07	24.94	24.72
*Nonessential amino acid* (*NEAA*, *%*)													
Alanine	4.37	4.57	4.67	2.73	5.67		3.50	3.57	3.72	3.40	3.34	3.61	3.80
Aspartic acid	6.36	6.69	3.22	6.97	3.74		4.92	4.80	4.62	5.03	5.17	4.82	4.67
Cysteine	1.24	0.90	1.30	0.41	1.70	0.06^c^	1.02	1.02	1.10	0.94	0.88	1.07	1.15
Glutamic acid	9.05	9.28	11.36	11.41	13.80		7.83	8.00	8.10	7.90	7.96	8.07	8.17
Glycine	4.26	4.39	1.43	2.62	1.68		3.14	3.03	2.90	3.10	2.97	3.04	2.92
Proline	3.14	3.05	5.14	3.30	6.47		2.46	2.63	2.75	2.48	2.51	2.69	2.87
Serine	2.95	3.05	2.72	3.28	3.26		2.38	2.46	2.54	2.41	2.45	2.50	2.59
Tyrosine	1.84	2.04	2.31	1.87	2.84		1.50	1.57	1.67	1.52	1.52	1.63	1.73
∑NEAA	33.21	33.97	32.15	32.59	39.16		26.75	27.08	27.4	26.78	26.8	27.43	27.9

^a,b,c^Data were obtained from Alam et al. [44], Forster and Ogata, and Alam et al. [44–46].

**Table 3:** Fatty acids (% of total fatty acids) profiles of the experimental diets replacing FM by various plant protein sources.

Fatty acid (%)	FM	JMM	CGM	SPC	CPC	Requirement	Experimental diets						
							Con	CGM25	CGM50	SPC25	SPC50	CPC25	CPC50
C12 : 0	0.07	0.05	0.01	0.53	0.01		0.04	0.02	0.00	0.07	0.09	0.02	0.00
C14 : 0	5.83	3.88	0.07	0.26	0.07		3.23	1.40	0.65	1.73	0.92	1.40	0.65
C15 : 0	0.35	0.48	0.02	0.09	0.02		0.21	0.11	0.05	0.14	0.09	0.11	0.05
C16 : 0	21.89	19.09	12.83	20.12	13.43		19.22	10.61	6.84	15.21	14.33	10.69	6.94
C17 : 0	0.45	0.79	0.08	0.28	0.08		0.84	0.50	0.39	0.69	0.51	0.49	0.38
C18 : 0	3.72	7.07	2.12	8.10	1.86		4.33	3.94	3.43	4.94	5.83	3.89	3.36
C20 : 0	0.29	0.29	0.49	0.67	0.43		0.25	0.28	0.35	0.35	0.46	0.32	0.34
C21 : 0	2.22	1.22		0.47			0.05	0.02	0.00	0.02	0.01	0.02	0.00
C22 : 0	0.15	0.23	0.33	0.82	0.39								
C23 : 0	0.07	0.08											
C24 : 0							1.32	0.74	0.51	0.90	0.71	0.70	0.52
∑SFA	35.01	33.18	15.95	31.34	16.29		29.49	16.88	11.71	23.15	22.24	16.94	11.72
C14 : 1*n*−5	0.10	0.09					0.07	0.05	0.04	0.04	0.05	0.03	0.04
C15 : 1*n*−7	0.11	0.17	0.02	0.05	0.01		0.04	0.02	0.01	0.03	0.02	0.02	0.01
C16 : 1*n*−7	6.53	5.43	0.23	0.19	0.23		3.67	1.68	0.82	2.05	1.11	1.68	0.82
C17 : 1*n*−7	1.08	1.07	0.05	0.19	0.07		0.62	0.29	0.15	0.38	0.22	0.30	0.15
C18 : 1*n*−9	15.47	21.41	25.88	33.07	24.50		22.94	28.11	30.03	29.21	31.83	27.78	24.53
C20 : 1*n*−9	0.48	0.61	0.01		0.01		1.43	0.73	0.37	0.89	0.50	0.73	0.37
C22 : 1*n*−9	0.07	0.15					0.43	0.27	0.15	0.33	0.20	0.27	0.15
C24 : 1*n*−9	0.63	0.46					0.28	0.12	0.05	0.15	0.07	0.12	0.05
∑MUFA	24.44	29.39	26.19	33.50	24.82		29.48	31.27	31.62	33.08	34.00	30.93	26.12
C18 : 2*n*−6	4.54	2.66	53.66	25.21	54.03		23.09	40.04	45.73	28.79	30.55	40.30	50.03
C18 : 3*n*−3	2.31	1.21	2.51	2.88	2.54		2.82	2.65	3.03	2.91	3.33	2.66	3.04
C18 : 3*n*−6	0.08	0.08	0.01		0.01		0.08	0.03	0.02	0.04	0.02	0.03	0.02
C20 : 2*n*−6	0.25	0.39	0.05	1.17	0.05		0.11	0.06	0.03	0.17	0.20	0.06	0.03
C20 : 3*n*−3	1.62	1.87	0.03	0.15	0.02		0.34	0.16	0.08	0.21	0.12	0.16	0.08
C20 : 3*n*−6				0.09	0.01		0.01	0.01	0.00	0.03	0.03	0.01	0.00
C20 : 4*n*−6	0.50	0.62					0.03	0.01	0.00	0.01	0.01	0.01	0.00
C20 : 5*n*−3	13.40	10.09					6.27	2.78	2.30	3.40	2.78	2.78	2.30
C22 : 2*n*−6							0.26	0.28	0.26	0.25	0.25	0.28	0.29
C22 : 5*n*−3	2.63	2.74											
C22 : 6*n*−3	12.15	13.99					4.79	4.37	4.17	4.89	4.60	4.36	4.67
∑*n*−3 HUFA^1^	29.80	28.69	0.03	0.24	0.03	7.62–9.52^a^	11.40	7.31	6.55	8.50	7.50	7.30	7.05
Unknown	3.08	3.78	1.60	5.66	2.23		3.23	1.46	1.05	3.07	1.87	1.48	1.70

^a^∑*n*−3 HUFA was obtained in Kim and Lee's [53] study.

**Table 4:** Survival (%), weight gain (g/fish), and specific growth rate (SGR) of olive flounder fed the experimental diets replacing different levels of FM with various plant protein sources for 56 days.

Experimental diets	Initial weight (g/fish)	Final weight (g/fish)	Survival (%)	Weight gain (g/fish)	SGR^1^ (%/day)
Con	18.0	87.0	100.0	69.0^a^	2.81^a^
CGM25	18.0	87.3	100.0	69.2^a^	2.82^a^
CGM50	18.0	83.9	100.0	65.8^bc^	2.75^bc^
SPC25	18.0	85.6	100.0	67.5^ab^	2.78^ab^
SPC50	18.0	82.3	100.0	64.3^c^	2.71^c^
CPC25	18.0	85.7	100.0	67.7^ab^	2.78^ab^
CPC50	18.0	84.3	100.0	66.3^bc^	2.76^bc^
Pooled SE			0.00	0.40	0.000
Main effect: replacement level					
25%				68.1^A^	2.79^A^
50%				65.5^B^	2.74^B^
Main effect: replacement source					
CGM				67.6^A^	2.78^A^
SPC				67.0^AB^	2.77^AB^
CPC				65.6^B^	2.75^B^
Two-way ANOVA					
Replacement level				*P* < 0.0001	*P* < 0.0001
Replacement source				*P* < 0.045	*P* < 0.033
Interaction				*P* > 0.189	*P* > 0.176

Values (means of triplicate ± SE) in the same column sharing the same superscript letter are not significantly different (*P* > 0.05). ^1^SGR (%/day) = ((Ln final weight of fish – Ln initial weight of fish) × 100)/days of feeding trial.

**Table 5:** Feed consumption (g/fish), feed efficiency ratio (FER), protein efficiency ratio (PER), protein retention (PR), condition factor (CF), viscerosomatic index (VSI), and hepatosomatic index (HSI) of olive flounder fed the experimental diets replacing different levels of FM with various plant protein source for 56 days.

Experimental diets	Feed consumption (g/fish)	FER^1^	PER^2^	PR^3^	CF^4^	VSI^5^	HSI^6^
Con	66.6^a^	1.04	1.85	33.6	0.93	6.16	1.72
CGM25	66.6^a^	1.04	1.85	33.9	0.97	6.14	1.72
CGM50	63.9^cd^	1.03	1.84	33.6	0.97	6.26	1.72
SPC25	66.1^ab^	1.02	1.82	33.4	0.95	6.02	1.72
SPC50	62.9^d^	1.02	1.82	33.3	0.94	6.27	1.72
CPC25	65.9^ab^	1.03	1.84	33.0	0.95	6.19	1.72
CPC50	64.8^bc^	1.02	1.83	33.6	0.95	6.23	1.73
Pooled SE	0.306	0.003	0.000	0.011	0.006	0.000	0.000
Main effect: replacement level							
25%	66.2^A^	1.03	1.84	34.1	0.96	6.12^AB^	1.72
50%	63.9^B^	1.02	1.83	34.1	0.96	6.26^A^	1.72
Main effect: replacement source							
CGM	65.6^A^	1.03	1.85	34.4	0.97	6.21	1.73
SPC	65.3^AB^	1.03	1.83	34.0	0.95	6.21	1.72
CPC	64.5^B^	1.02	1.82	33.9	0.95	6.20	1.72
Two-way ANOVA							
Replacement level	*P* < 0.0001	*P* > 0.455	*P* > 0.508	*P* > 0.853	*P* > 0.879	*P* < 0.037	*P* > 0.606
Replacement source	*P* < 0.030	*P* > 0.324	*P* > 0.265	*P* > 0.211	*P* > 0.436	*P* > 0.599	*P* > 0.838
Interaction	*P* < 0.012	*P* > 0.910	*P* > 0.984	*P* > 0.222	*P* > 0.925	*P* > 0.344	*P* > 0.918

Values (means of triplicate ± SE) in the same column sharing the same superscript letter are not significantly different (*P* > 0.05). ^1^Feed efficiency ratio (FER) = weight gain of fish/feed consumption. ^2^Protein efficiency ratio (PER) = weight gain of fish/protein consumption. ^3^Protein retention (PR, %) = protein gain of fish × 100/protein consumption. ^4^Condition factor (CF, g/cm^3^) = total weight of fish (g) × 100/total length of fish (cm)^3^. ^5^Viscerosomatic index (VSI, %) = viscera weight × 100/total weight of fish. ^6^Hepatosomatic index (HSI, %) = liver weight × 100/total weight of fish.

**Table 6:** Proximate composition (%) of the whole body of olive flounder-fed experimental diets replacing FM with various plant protein sources for 56 days.

Experimental diets	Moisture	Crude protein	Crude lipid	Ash
Con	71.0	18.3	3.3	4.2
CGM25	70.9	18.3	3.5	4.4
CGM50	71.3	18.3	3.6	4.4
SPC25	71.6	18.3	3.4	3.9
SPC50	71.7	18.3	3.7	4.5
CPC25	71.7	18.0	3.4	4.3
CPC50	71.0	18.4	3.2	3.7
Pooled SE	0.01	0.00	0.00	0.00
Main effect: replacement level				
25%	71.4	18.2	3.4	4.2
50%	71.3	18.3	3.5	4.2
Main effect: replacement source				
CGM	71.1	18.3	3.5	4.4
SPC	71.6	18.3	3.6	4.2
CPC	71.3	18.2	3.3	4.0
Two-way ANOVA				
Replacement level	*P* > 0.348	*P* > 0.824	*P* > 0.195	*P* > 0.744
Replacement source	*P* > 0.526	*P* > 0.285	*P* > 0.652	*P* > 0.348
Interaction	*P* > 0.534	*P* < 0.035	*P* > 0.803	*P* > 0.223

Values (means of triplicate ± SE) in the same column sharing the same superscript letter are not significantly different (*P* > 0.05).

**Table 7:** Amino acid profiles (wet weight %) of the whole body of olive flounder fed the experimental diets replacing FM with various plant protein sources for 56 days.

Experimental diets									Main effect: replacement level		Main effect: replacement source			Two-way ANOVA		
	Con	CGM25	CGM50	SPC25	SPC50	CPC25	CPC50	Pooled SE	25%	50%	CGM	SPC	CPC	Replacement level	Replacement source	Interaction
*Essential amino acid* (*%*)																
Arginine	1.2	1.2	1.2	1.1	1.2	1.2	1.1	0.01	1.2	1.2	1.2	1.1	1.2	*P* > 0.455	*P* > 0.653	*P* > 0.584
Histidine	0.4	0.4	0.4	0.4	0.4	0.4	0.4	0.01	0.4	0.4	0.4	0.4	0.4	*P* > 0.868	*P* > 0.979	*P* > 0.708
Isoleucine	0.7	0.7	0.7	0.7	0.7	0.7	0.7	0.01	0.7	0.7	0.7	0.7	0.7	*P* > 0.904	*P* > 0.952	*P* > 0.795
Leucine	1.3	1.3	1.3	1.3	1.3	1.4	1.3	0.01	1.3	1.3	1.3	1.3	1.4	*P* > 0.828	*P* > 0.756	*P* > 0.810
Lysine	1.6	1.5	1.6	1.6	1.6	1.6	1.6	0.01	1.6	1.6	1.6	1.6	1.6	*P* > 0.755	*P* > 0.509	*P* > 0.882
Methionine	0.5	0.5	0.5	0.5	0.5	0.5	0.5	0.01	0.5	0.5	0.5	0.5	0.5	*P* > 0.387	*P* > 0.695	*P* > 0.972
Phenylalanine	0.7	0.7	0.7	0.7	0.7	0.7	0.7	0.01	0.7	0.7	0.7	0.7	0.7	*P* > 0.244	*P* > 0.995	*P* > 0.714
Threonine	0.8	0.8	0.8	0.8	0.8	0.8	0.8	0.01	0.8	0.8	0.8	0.8	0.8	*P* > 0.838	*P* > 0.620	*P* > 0.836
Tryptophan	0.1	0.1	0.1	0.1	0.1	0.1	0.1	0.00	0.1	0.1	0.1	0.1	0.1	*P* > 0.533	*P* > 0.875	*P* > 0.875
Valine	0.9	0.9	0.9	0.8	0.9	0.9	0.9	0.01	0.9	0.9	0.9	0.9	0.9	*P* > 0.614	*P* > 0.707	*P* > 0.675
*Nonessential amino acid* (*%*)																
Alanine	1.4	1.5	1.4	1.4	1.4	1.4	1.4	0.02	1.5	1.4	1.4	1.4	1.4	*P* > 0.198	*P* > 0.937	*P* > 0.627
Aspartic acid	1.7	1.8	1.7	1.7	1.8	1.8	1.7	0.02	1.8	1.7	1.7	1.7	1.8	*P* > 0.297	*P* > 0.290	*P* > 0.092
Cysteine	0.2	0.2	0.2	0.2	0.2	0.2	0.2	0.00	0.2	0.2	0.2	0.2	0.2	*P* > 0.399	*P* > 0.985	*P* > 0.628
Glutamic acid	2.6	2.6	2.6	2.6	2.6	2.6	2.7	0.03	2.6	2.6	2.7	2.6	2.6	*P* > 0.697	*P* > 0.392	*P* > 0.665
Glycine	1.5	1.7	1.6	1.4	1.4	1.7	1.5	0.03	1.6	1.5	1.7^A^	1.4^B^	1.6^AB^	*P* > 0.699	*P* < 0.024	*P* > 0.540
Proline	0.9	0.9	0.9	0.9	0.9	0.9	0.9	0.02	0.9	0.9	0.9	0.9	0.9	*P* > 0.192	*P* > 0.917	*P* > 0.825
Serine	0.9	0.9	0.8	0.9	0.9	0.9	0.9	0.01	0.9	0.9	0.9	0.9	0.9	*P* > 0.302	*P* > 0.915	*P* > 0.484
Tyrosine	0.4	0.5	0.5	0.5	0.5	0.5	0.5	0.01	0.5	0.5	0.5	0.5	0.5	*P* > 0.926	*P* > 0.581	*P* > 0.653

Values (means of triplicate ± SE) in the same row sharing the same superscript letter are not significantly different (*P* > 0.05).

**Table 8:** Fatty acid profiles (% of total fatty acids) of the whole body of olive flounder fed the experimental diets replacing FM with various plant protein sources for 56 days.

Experimental diets									Main effect: replacement level		Main effect: replacement source			Two-way ANOVA		
	Con	CGM25	CGM50	SPC25	SPC50	CPC25	CPC50	Pooled SE	25%	50%	CGM	SPC	CPC	Replacement level	Replacement source	Interaction
C12 : 0	0.1	0.1	0.1	0.1	0.1	0.1	0.1	0.01	0.1	0.1	0.1	0.1	0.1	*P* > 0.565	*P* > 0.296	*P* > 0.534
C14 : 0	3.7	3.6	3.7	3.7	3.6	3.7	3.7	0.03	3.7	3.7	3.7	3.7	3.7	*P* > 0.999	*P* > 0.777	*P* > 0.700
C15 : 0	0.3	0.3	0.3	0.2	0.3	0.3	0.3	0.01	0.3	0.3	0.3	0.3	0.3	*P* > 0.285	*P* > 0.159	*P* > 0.212
C16 : 0	17.3	17.3	17.4	17.2	17.3	17.3	17.3	0.04	17.3	17.3	17.3	17.3	17.3	*P* > 0.847	*P* > 0.795	*P* > 0.904
C17 : 0	0.9	0.9	0.9	0.9	0.9	0.9	0.9	0.01	0.9	0.9	0.9	0.9	0.9	*P* > 0.875	*P* > 0.813	*P* > 0.850
C18 : 0	4.1	4.1	4.1	4.1	4.2	4.1	4.1	0.04	4.1	4.1	4.1	4.1	4.1	*P* > 0.999	*P* > 0.898	*P* > 0.829
C20 : 0	0.4	0.3	0.4	0.4	0.4	0.4	0.4	0.01	0.4	0.4	0.4	0.4	0.4	*P* > 0.999	*P* > 0.391	*P* > 0.750
C21 : 0	0.1	0.1	0.1	0.1	0.1	0.1	0.1	0.01	0.1	0.1	0.1	0.1	0.1	*P* > 0.935	*P* > 0.747	*P* > 0.718
C24 : 0	1.9	1.9	1.9	1.8	1.9	1.9	1.9	0.01	1.9	1.9	1.9	1.9	1.9	*P* > 0.557	*P* > 0.062	*P* > 0.966
∑SFA	28.8	28.7	28.9	28.6	28.7	28.8	28.7	0.05	28.7	28.8	28.8	28.7	28.8	*P* > 0.731	*P* > 0.651	*P* > 0.546
C14 : 1*n*−5	0.1	0.1	0.1	0.1	0.1	0.1	0.1	0.01	0.1	0.1	0.1	0.1	0.1	*P* > 0.626	*P* > 0.809	*P* > 0.979
C15 : 1*n*−7	0.3	0.3	0.3	0.3	0.3	0.3	0.3	0.01	0.3	0.3	0.3	0.3	0.3	*P* > 0.972	*P* > 0.881	*P* > 0.984
C16 : 1*n*−7	4.5	4.5	4.5	4.5	4.5	4.5	4.5	0.03	4.5	4.5	4.5	4.5	4.5	*P* > 0.942	*P* > 0.998	*P* > 0.980
C17 : 1*n*−7	0.7	0.7	0.7	0.7	0.7	0.7	0.7	0.05	0.7	0.7	0.7	0.7	0.7	*P* > 0.765	*P* > 0.882	*P* > 0.933
C18 : 1*n*−9	24.	24.2	24.2	24.3	24.2	24.3	24.3	0.01	24.3	24.2	24.2	24.3	24.3	*P* > 0.669	*P* > 0.773	*P* > 0.814
C20 : 1*n*−9	1.8	1.8	1.8	1.8	1.8	1.8	1.8	0.03	1.8	1.8	1.8	1.8	1.8	*P* > 0.930	*P* > 0.976	*P* > 0.902
C22 : 1*n*−9	1.3	1.3	1.3	1.3	1.3	1.3	1.3	0.01	1.3	1.3	1.3	1.3	1.3	*P* > 0.930	*P* > 0.926	*P* > 0.995
C24 : 1*n*−9	0.2	0.3	0.2	0.2	0.3	0.2	0.2	0.01	0.2	0.2	0.2	0.2	0.2	*P* > 0.703	*P* > 0.908	*P* > 0.908
∑MUFA	33.2	33.1	33.1	33.2	33.1	33.2	33.2	0.07	33.2	33.1	33.1	33.2	33.2	*P* > 0.794	*P* > 0.908	*P* > 0.945
C18 : 2*n*−6	18.2	18.1	18.2	18.2	18.1	18.2	18.1	0.08	18.2	18.1	18.1	18.2	18.2	*P* > 0.865	*P* > 0.984	*P* > 0.921
C18 : 3*n*−3	1.7	1.7	1.7	1.7	1.7	1.7	1.7	0.03	1.7	1.7	1.7	1.7	1.7	*P* > 0.883	*P* > 0.980	*P* > 0.960
C18 : 3*n*−6	0.2	0.2	0.2	0.2	0.2	0.2	0.2	0.01	0.2	0.2	0.2	0.2	0.2	*P* > 0.403	*P* > 0.579	*P* > 0.455
C20 : 2*n*−6	0.6	0.6	0.5	0.5	0.6	0.6	0.5	0.01	0.6	0.5	0.5	0.5	0.6	*P* > 0.793	*P* > 0.918	*P* > 0.536
C20 : 3*n*−3	0.8	0.8	0.8	0.8	0.8	0.8	0.8	0.01	0.8	0.8	0.8	0.8	0.8	*P* > 0.565	*P* > 0.959	*P* > 0.951
C20 : 3*n*−6	0.03	0.01	0.01	0.04	0.05	0.03	0.04	0.01	0.04	0.04	0.04	0.04	0.04	*P* > 0.589	*P* > 0.783	*P* > 0.875
C20 : 4*n*−6	0.2	0.2	0.2	0.2	0.2	0.2	0.2	0.01	0.2	0.2	0.2	0.2	0.2	*P* > 0.717	*P* > 0.991	*P* > 0.283
C20 : 5*n*−3	6.3	6.4	6.4	6.4	6.4	6.4	6.4	0.04	6.4	6.4	6.4	6.4	6.4	*P* > 0.837	*P* > 0.964	*P* > 0.993
C22 : 2*n*−6	0.3	0.3	0.4	0.3	0.3	0.3	0.3	0.01	0.3	0.3	0.3	0.3	0.3	*P* > 0.420	*P* > 0.484	*P* > 0.424
C22 : 6*n*−3	6.2	6.4	6.4	6.3	6.2	6.2	6.3	0.03	6.3	6.3	6.4	6.2	6.3	*P* > 0.897	*P* > 0.255	*P* > 0.666
∑*n*−3 HUFA	13.3	13.5	13.5	13.4	13.3	13.4	13.4	0.05	13.4	13.4	13.5	13.3	13.4	*P* > 0.922	*P* > 0.452	*P* > 0.882
Unknown	3.6	3.6	3.2	3.6	3.8	3.4	3.6	0.12	3.5	3.5	3.4	3.7	3.5	*P* > 0.954	*P* > 0.760	*P* > 0.699

Values (means of triplicate ± SE) in the same row sharing the same superscript letter are not significantly different (*P* > 0.05).

**Table 9:** Hematological parameters of olive flounder-fed experimental diets replacing different levels of FM with various plant protein sources for 56 days.

Experimental diets	AST (IU/L)	ALT (IU/L)	ALP (IU/L)	TBL (mg/dL)	TCL (mg/dL)	TGL (mg/dL)	TPT (g/dL)	ABM (g/dL)
Con	17.7	3.7	107.9	0.6	310.6	318.7	4.6	1.7
CGM25	16.8	4.2	111.7	0.6	316.0	276.6	4.3	2.1
CGM50	17.0	6.1	104.8	0.5	339.7	291.8	4.4	1.9
SPC25	18.0	6.2	111.0	0.6	277.4	287.2	4.3	1.9
SPC50	16.2	4.1	110.8	0.6	268.3	274.8	5.1	2.2
CPC25	16.4	3.4	105.4	0.6	315.9	292.8	4.1	2.0
CPC50	14.6	5.0	107.0	0.6	271.8	319.1	4.2	1.9
Pooled SE	0.58	0.36	1.49	0.03	7.36	7.08	0.11	0.08
Main effect: replacement level								
25%	15.9	5.1	109.4	0.6	303.1	285.5	4.2	2.0
50%	17.1	4.6	107.5	0.5	293.3	295.2	4.6	2.0
Main effect: replacement source								
CGM	16.9	5.2	108.2	0.5	327.8^A^	284.2	4.4	2.0
SPC	17.1	5.2	110.9	0.6	272.9^B^	281.0	4.7	2.0
CPC	15.5	4.2	106.2	0.6	293.8^AB^	306.0	4.1	1.9
Two-way ANOVA								
Replacement level	*P* > 0.332	*P* > 0.631	*P* > 0.435	*P* > 0.775	*P* > 0.414	*P* > 0.480	*P* > 0.191	*P* > 0.947
Replacement source	*P* > 0.492	*P* > 0.651	*P* > 0.294	*P* > 0.919	*P* < 0.008	*P* > 0.288	*P* > 0.234	*P* > 0.968
Interaction	*P* > 0.699	*P* > 0.189	*P* > 0.326	*P* > 0.919	*P* > 0.099	*P* > 0.495	*P* > 0.406	*P* > 0.455

Values (means of triplicate ± SE) in the same column sharing the same superscript letter are not significantly different (*P* > 0.05).

**Table 10:** Lysozyme and superoxide dismutase (SOD) activities of olive flounder-fed the experimental diets replacing different levels of FM with various plant protein sources for 56 days.

Experimental diets	Lysozyme activity (U/mL)	SOD activity (%)
Con	393.2	72.0
CGM25	450.4	73.6
CGM50	416.0	72.8
SPC25	394.4	72.5
SPC50	390.1	72.8
CPC25	418.0	72.3
CPC50	381.6	71.5
Pooled SE	0.00	8.29
Main effect: replacement level		
25%	420.9	72.8
50%	395.9	72.4
Main effect: replacement source		
CGM	433.2	73.2
SPC	392.2	72.6
CPC	399.8	71.9
Two-way ANOVA		
Replacement level	*P* > 0.226	*P* > 0.377
Replacement source	*P* > 0.235	*P* > 0.110
Interaction	*P* > 0.761	*P* > 0.562

Values (means of triplicate ± SE) in the same column sharing the same superscript letter are not significantly different (*P* > 0.05).

## Data Availability

Data are available on request from the authors.
